# Supplementary material for: What does it take to learn the rules of RNA base pairing? A lot less than you may think
Source: bioRxiv. 2025 Aug 2:2025.07.31.668042. Preprint. [Version 1] doi: 10.1101/2025.07.31.668042 (PMC12324431; doi:10.1101/2025.07.31.668042)
Supplement: Supplement 1 [file media-1.gz › supplemental_material/src/rscape_v2.6.0.tar.gz!/rscape_v2.6.0/lib/infernal/Manuscripts/banded-cyk/figs/roc.pdf]

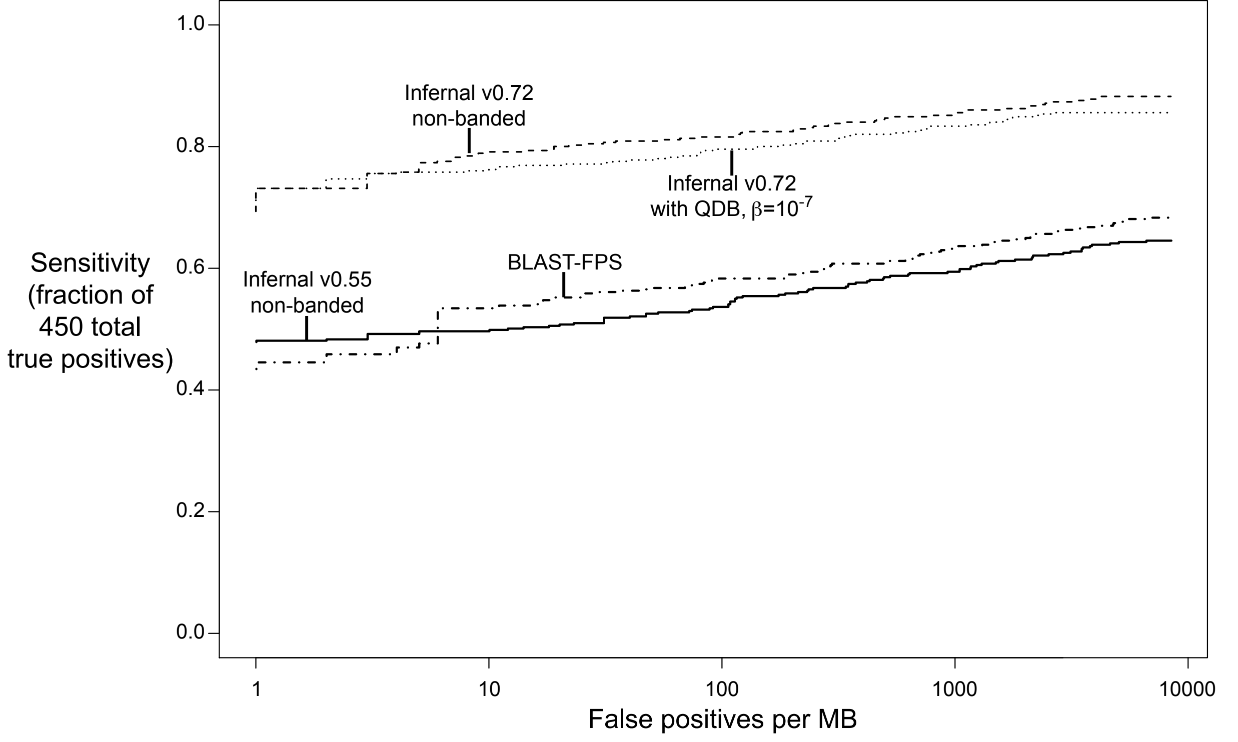
Infernal v0.72
non-banded
Infernal v0.72
with QDB, β=10^-7
BLAST-FPS
Infernal v0.55
non-banded
Sensitivity
(fraction of
450 total
true positives)
1.0
0.8
0.6
0.4
0.2
0.0
1
10
100
1000
10000
False positives per MB
